# O02 Antibiotic prescribing practices in primary healthcare in sub-Saharan Africa: a systematic review and our experience on the use of telemedicine in Zambia

**DOI:** 10.1093/jacamr/dlac003.001

**Published:** 2022-02-16

**Authors:** Moe Takenoshita, David Hettle, Graziella Quattrocchi

**Affiliations:** The Virtual Doctors’ charity, Brighton, UK; University of Cambridge Medical School, Cambridge, UK; The Virtual Doctors’ charity, Brighton, UK; North Bristol NHS Trust, Bristol, UK; The Virtual Doctors’ charity, Brighton, UK; North Middlesex University Hospital, London, UK

## Abstract

**Background:**

Antimicrobial resistance (AMR) presents a challenge to clinicians globally, particularly in low- and middle-income countries (LMICs) which often have a high prevalence of infectious diseases, coupled with limited diagnostic, treatment and surveillance capabilities.^1,2^ To date, there is limited information on antimicrobial prescribing in sub-Saharan Africa, particularly in rural clinics,^3^ where systematic data gathering can be impacted by the resource-poor nature of these settings. The Virtual Doctors charity (VDrs)^4^ uses a bespoke smartphone app to give remote telemedical advice to clinicians throughout rural Zambia. This approach links primary healthcare workers with volunteer doctors, predominantly based in the UK. We propose that this approach may be a valuable tool to promote antimicrobial stewardship (AMS) in these locations, and consequently that telemedicine can play a key role in preventing a future AMR pandemic.

**Methods:**

We carried out a systematic review to investigate current antibiotic prescribing practices in LMICs within sub-Saharan Africa, specifically the indications for use, choice of antimicrobial, and prescribing rates. Three Africa-specific databases, EMBASE, MEDLINE, Cochrane Library, Web of Science, Scopus, CINAHL, IRIS and Global Health databases were searched. English language studies evaluating antimicrobial use in primary care in LMICs in sub-Saharan Africa published between January 2010 and July 2021 were included. Data on the prevalence of antibiotic prescription, choice of agent and appropriateness was collated. We then reviewed anonymized prescribing data from the VDrs database, investigating antibiotic prescribing in cases over a period between January 2019 and June 2020. We excluded non-adult (<18 years old) patients, and reviewed cases that had been coded as ‘InfectiousDiseases’, ‘RespiratoryMedicine’ or ‘MedicineGeneral’ to identify those in which antibiotics were prescribed acutely and the impact of consultations on antibiotic prescriptions.

**Results:**

Systematic review: 29 eligible studies from nine countries were identified, with 18 (62%) undertaken in urban settings and 7 (24%) encompassing rural settings. The prevalence of antibiotic prescription was 32.7%–84.4% (median 55.2%, IQR 44.5%–67.7%), with respiratory and genitourinary infections the most common indications. The most prescribed antibiotics were β-lactams (primarily amoxicillin), then quinolones and metronidazole. Only two studies assessed the appropriateness of prescriptions, with 13%–36.4% found to be inappropriate. Retrospective cohort analysis of VDrs database: 502 cases were reviewed. 154 (30.7%) were receiving 1–3 antibiotic agents at the time of consultation. The most prescribed agents were metronidazole and amoxicillin. Following consultation, 53 patients (10.5%) were initiated on antibiotics. Of those already receiving antibiotics, 29.2% had these actively continued, and 45.5% had antibiotics rationalized or stopped based on volunteers’ advice.

**Conclusions:**

Our systematic review confirms high rates of antimicrobial prescription in LMICs in sub-Saharan Africa, with a significant proportion being inappropriate. However, data are limited, and mainly from urban settings, hindering the generalizability of results. Our analysis of VDrs data reveals that 45.5% of antibiotic prescriptions were rationalized through our telemedicine-based approach. The VDrs model aims not only to deliver case-based guidance to rural healthcare practitioners, but also provide long-term educational gains to enhance local and global health. We suggest that the appropriate use of telemedicine can have short- and long-term benefits for AMS in LMICs.
